# Profiling neuronal ion channelopathies with non-invasive brain imaging and dynamic causal models: Case studies of single gene mutations

**DOI:** 10.1016/j.neuroimage.2015.08.057

**Published:** 2016-01-01

**Authors:** Jessica R. Gilbert, Mkael Symmonds, Michael G. Hanna, Raymond J. Dolan, Karl J. Friston, Rosalyn J. Moran

**Affiliations:** a Virginia Tech Carilion Research Institute, Roanoke, VA 24016, USA; b Nuffield Department of Clinical Neurosciences, John Radcliffe Hospital, Oxford University, Oxford OX3 9DU, UK; c Institute of Neurology, University College London, 9 Queen Square, London WC1N 3BG, UK; d Max Planck UCL Centre for Computational Psychiatry and Ageing Research, Russell Square House, 10-12 Russell Square, London WC1B 5EH, UK; e Wellcome Trust Centre for Neuroimaging, Institute of Neurology, University College London, 12 Queen Square, London WC1N 3BG, UK; f Bradley Department of Electrical and Computer Engineering, Virginia Polytechnic Institute and State University, Blacksburg, VA 24061, USA

**Keywords:** Channelopathies, Dynamic causal modeling, Magnetoencephalography, Ion channel signaling, Biophysical models

## Abstract

Clinical assessments of brain function rely upon visual inspection of electroencephalographic waveform abnormalities in tandem with functional magnetic resonance imaging. However, no current technology proffers in vivo assessments of activity at synapses, receptors and ion-channels, the basis of neuronal communication. Using dynamic causal modeling we compared electrophysiological responses from two patients with distinct monogenic ion channelopathies and a large cohort of healthy controls to demonstrate the feasibility of assaying synaptic-level channel communication non-invasively. Synaptic channel abnormality was identified in both patients (100% sensitivity) with assay specificity above 89%, furnishing estimates of neurotransmitter and voltage-gated ion throughput of sodium, calcium, chloride and potassium. This performance indicates a potential novel application as an adjunct for clinical assessments in neurological and psychiatric settings. More broadly, these findings indicate that biophysical models of synaptic channels can be estimated non-invasively, having important implications for advancing human neuroimaging to the level of non-invasive ion channel assays.

## Highlights

• Dynamic causal modeling (DCM) for M/EEG includes ion channel parameter estimates.
• Parameter estimates from patients with monogenic ion channelopathies were compared.
• Synaptic channel abnormality was identified in patients, with specificity above 89%.
• DCM could serve as a platform for non-invasively assaying brain molecular dynamics.

## Introduction

The balanced flow of ions through synapses is integral to the stability and control of neuronal firing and information transmission in the brain. Abnormalities of ion channels and synaptic function are thought to underlie a range of neurological presentations including seizures, migraine and movement disorders (Catterall et al., 2008), and inform pharmacological treatment strategies (Yogeeswari et al., 2004). Furthermore, a growing body of evidence in psychiatry suggests alterations in simple mechanistic principles, like the ratio of neocortical excitation to inhibition or long-range to local synaptic transmission, could underlie complex disorders including social dysfunction (Yizhar et al., 2011), autism (Rubenstein and Merzenich, 2003) and schizophrenia (Jardri and Deneve, 2013). In animal models of these diseases, tools like optogenetics provide a means to manipulate synaptic transmission, providing a platform to test putative pathophysiological mechanisms in neural circuits (Tye and Deisseroth, 2012). However, there are no current technologies for non-invasively measuring brain function at this level in humans. While limited assessments of neurotransmitter and synaptic receptor levels are feasible with magnetic resonance spectroscopy (Dager et al., 2008) and positron emission tomography (Lee and Farde, 2006), these techniques do not directly measure neuronal function and can be applied only to a limited set of molecules. Here we describe how magnetic event-related fields (ERFs), measured at superconducting sensors around the head, can be fit to a biophysical model of neural circuits to recover potential probabilistic markers of an individual's synaptic function.

In this work, we utilize the specificity imparted by single-gene mutation neurological channelopathies (Catterall et al., 2008, Hanna, 2006, Kullmann, 2010) to assess the capability of our model-based assay in the context of magnetoencephalography (MEG). These signals are a close analogue of the electroencephalogram (EEG) and provide proof-of-principle for use in clinical settings, where EEG is widely available. Channelopathies, by virtue of their diverse clinical presentations (Catterall et al., 2008), illustrate how the functional consequence of particular ligand or voltage-gated ion channel dysfunction are neither easily predictable nor strictly amenable to diagnosis via clinical examination (Helbig et al., 2008). For example, patients with monogenic causes of epilepsy, such as generalized epilepsy with febrile seizures caused by mutations in neuronal sodium channels, can present with seizures of variable phenotypes and severities at different ages (Singh et al., 1999). In addition, genetic channel mutations can be partially penetrant, leading to differential interaction characteristics with various genes or with the environment (Kullmann, 2010). Outside of acquired (e.g., autoimmune) and single gene mutations, diagnosis of polygenic channel dysfunction (e.g., associated with idiopathic epilepsy (Cannon, 2006, Hanna, 2006)) using genome or exome sequencing suffers from a complex landscape of allelic risk; where non-affected individuals can harbor mutations in known or suspected epilepsy genes (Klassen et al., 2011). Therefore, patients with primary effectors from “mixed”, as well as classical single-gene channelopathies could benefit diagnostically and prognostically from the in situ characterization of pre and postsynaptic neuronal cell dynamics. Indeed, in silico computational models of channel variation have been proposed as a crucial bridge between genetics and disease risk or drug responsivity (Klassen et al., 2011).

Our work – based on dynamic causal modeling (DCM) – goes a step further by providing a biophysical model of currents produced by interacting ion channels which are then matched to measurable electromagnetic signals. This means that empirical data can be used to test competing models — and the winning model can be optimized for a given individual. DCM was originally designed as an analysis framework for imaging network-level communication in functional magnetic resonance imaging (Friston et al., 2003) and has been developed to infer the synaptic basis of measured electrophysiological signals like those from MEG, EEG and intracranial local field potentials (David et al., 2006). Previously, DCM has been applied in healthy human participants to assess putative synaptic changes induced by pharmacological agents like l-DOPA (Moran et al., 2011c) and propofol (Boly et al., 2012). It has also been used in patients to test the contribution of long-range and regionally-specific connections to the vegetative state (Boly et al., 2011).

**Fig. 1:**
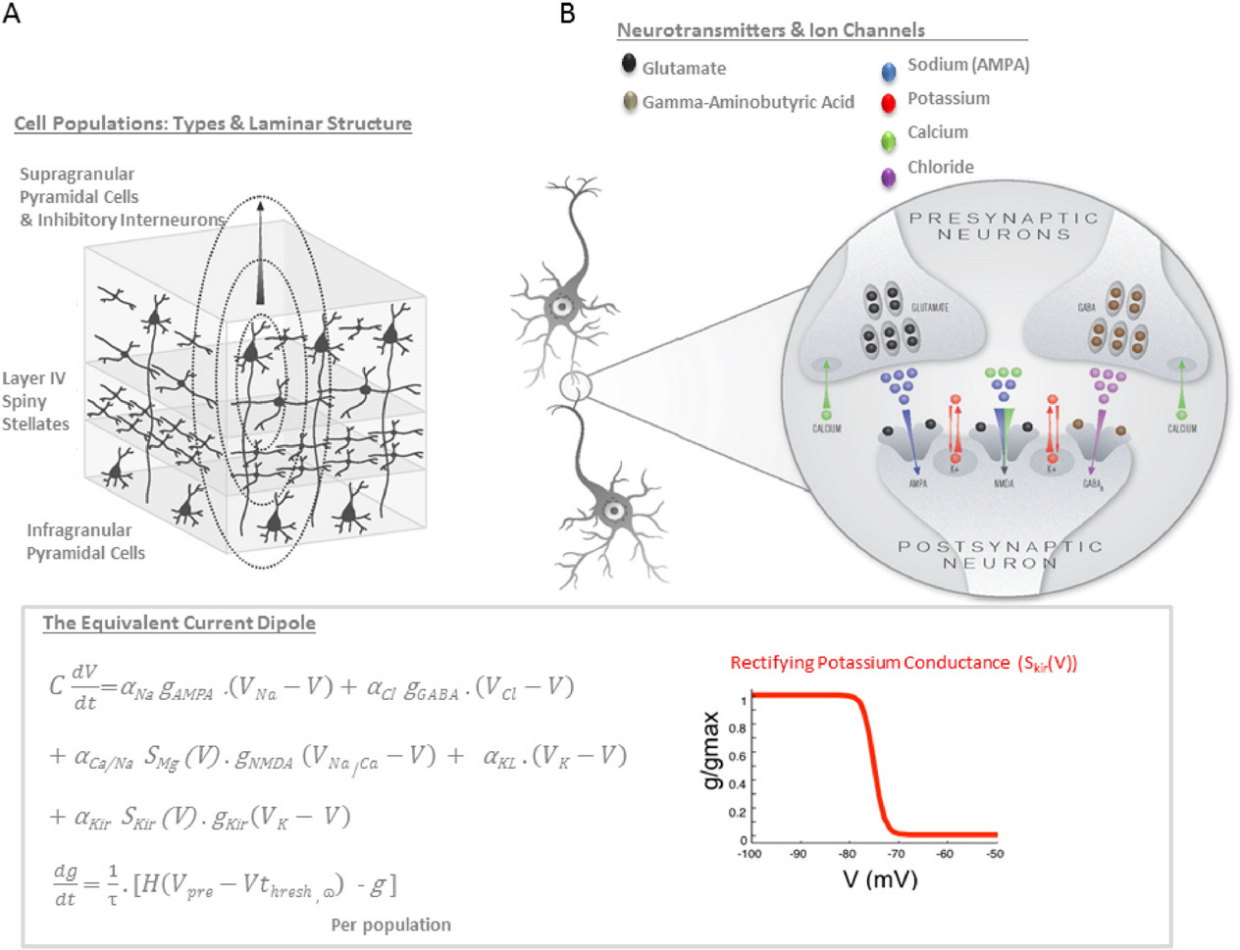
Properties of the dynamic causal model. A. For the DCM, three populations of neurons are used to model the activity of a given source of electromagnetic signals. These populations, including spiny stellate cells, pyramidal cells and inhibitory interneurons, are associated with cortical layers by virtue of their intrinsic connectivity — where layer IV stellates receive forward inputs, and supra and infragranular pyramidal cells and inhibitory interneurons are the targets of backward connections. B. The population dynamics are approximated by a mean field reduction, where average channel properties control the synaptic activity at each population of cells (Marreiros et al., 2009). This synaptic activity represents neurotransmitter and voltage-gated ion channels with distinct dynamics. C. The channels include a glutamatergic AMPA-mediated sodium channel, a glutamatergic NMDA-mediated sodium and calcium channel, a GABA_A_ mediated chloride channel, a leak potassium channel and an inward rectifying potassium channel. C. The dynamics at each population are formally described by a set of coupled differential equations where changes in postsynaptic depolarization (dV/dt) are governed by the dynamics of these channels with weights α and time constants τ. Reversal potential (V_ion_) determines the direction of current flow. Channel conductance (*g*) have time constants τ, and are dependent on presynaptic firing *H*, which is a function of presynaptic membrane potential (*V_pre_*) and the threshold potential (*V_thresh_*). The inward rectifying potassium channel (right, in red) is gated to produce maximal conductance at hyperpolarized states.

Here, we test sensitivity and specificity of the synaptic ion channel inferences available through electrophysiological DCM, utilizing data from two cases of single-gene mutation channelopathies. In order to test these particular patients we augmented a conductance-based neural mass model (Moran et al., 2011c) of regionally specific sources (Garrido et al., 2007) to include ligand-gated sodium, calcium, and chloride channels — as well as voltage-gated potassium and calcium channels (Fig. 1). This augmented model was used to explain auditory-evoked ERFs produced by 94 healthy control participants and 2 patients with known mutations causing loss-of-function in the inward-rectifying potassium channel gene KCNJ2 and in the voltage-gated presynaptic calcium channel gene CACNA1A. Our hope was to show a selective abnormality in the inferred model parameters encoding channel function that was consistent with the known channelopathies.

## Materials & methods

### Participants

Healthy control cohort: Ninety-four healthy control subjects participated in the experiment. Subjects ranged in age from 20 to 83 (Mean = 46.8 years), with 42 male participants. All patients were free from neurological or psychiatric disorders. Patients were recruited from the metropolitan London region.

Two patients were recruited through the National Hospital for Neurology and Neurosurgery in London. Patient selection was based on a previous diagnostic confirmation of single-gene mutations. Patient 1 (female, 54 years) had a mutation of gene KCNJ2. This gene encodes subunit Kir2.1, which is a potassium inward-rectifying channel (Kullmann, 2010). This patient presented with Andersen–Tawil syndrome, having a primary phenotype of periodic paralysis. Patient 2 (female, 45 years) had a mutation of gene CACNA1A. This gene encodes subunit α1A of Cav2.1, which impacts calcium neurotransmitter release at synapses (Kullmann, 2010). This patient presented with episodic ataxia (EA type 2). All participants were paid for their participation and provided written and informed consent to all procedures, which were conducted in accordance with the Declaration of Helsinki (1991). Protocols were approved by the South-East Strategic Health Authority Regional NHS Ethics Committee.

### MEG acquisition & preprocessing

MEG recordings were collected using a 275-channel CTF system with SQUID-based axial gradiometers (VSM MedTech Ltd., Couquitlam, BC, Canada). For each participant, two recording sessions were obtained, with a small rest period between sessions. Head localization was performed prior to each recording session using three energized electrical coils attached to fiducial locations (left and right pre-auricular, nasion). During each session, a passive listening auditory oddball paradigm was administered in which standard (i.e., frequent; 88%) tones of duration 70 msec (10 msec rise/fall times) and frequency 500 Hz were pseudo-randomly interspersed with oddball (i.e., infrequent; 12%) tones of frequency 800 Hz. A total of 400 trials were presented per session with an inter-trial interval fixed at 1100 msec. Participants were instructed to remain still while fixating to a central cross presented on a screen in front of them during the sessions. This paradigm was found to elicit a prominent mismatch negativity (MMN) response in the healthy controls, computed as the difference between standard and oddball evoked fields at around 150 msec peristimulus time (Moran et al., 2014).

MEG data were first filtered off-line, band-passed from 0.5–30 Hz, then down-sampled to 200 Hz. ERFs were computed for each participant by first epoching from − 100 msec to 350 msec peristimulus time. Epochs were baseline corrected against − 100 msec to 0 msec peristimulus time, and artifact corrected using a peak-to-peak threshold of 5 pF. Finally, the data were averaged to obtain ERFs. For preprocessing we used the analysis routines available in the academic freeware SPM8 (Wellcome Trust Centre for Neuroimaging, London, UK, http://www.fil.ion.ucl.ac.uk/spm/).

### Model of ion channels

We used an augmented neural mass model (NMM) that forms part of the DCM modeling options (listed as the NMM (Moran et al., 2013a)). This is a Morris–Lecar like model (Morris and Lecar, 1981) designed to generate neuronal population dynamics from large cell assemblies (Marreiros et al., 2009). This model mimics the net synaptic activity at an active neuronal population, which can be summarized as an equivalent current dipole (Fig. 1A). In our previous work (Moran et al., 2011c), we included active neurotransmitter-specific synaptic responses including ligand-gated excitatory (Na^+^) and inhibitory (Cl^−^) ion flow mediated by fast glutamatergic (AMPA) and GABAergic (GABA_A_) receptors respectively, as well as sodium (Na^+^) and calcium (Ca^2 +^) currents through voltage-gated NMDA receptors. For the present study, our model included these channels as they have been shown to accurately recapitulate the dynamics of the auditory evoked response (Moran et al., 2014). We further included a passive inward-rectifying potassium (K^+^) channel with a nonlinear membrane depolarization dependency (Figs. 1B, C) in order to capture the potential channel dysfunction in Patient 1. This channel (KIR) was modeled after Nisenbaum & Wilson (Nisenbaum and Wilson, 1995), with a sigmoidal switch function that scaled the conductance to be maximal at hyperpolarized states, zero at depolarized membrane potentials and 50% at − 75 mV (Fig. 1C). Dynamics were governed by ionic reversal potentials V*_ion_* with prior values: V*_NA_* = 60 mV, V*_NA/CA_* = 60 mV, V*_Cl_* = − 90 mV and V*_K_* = − 70 mV. Channels were considered with particular receptor or voltage-dependent time constants τ*_channel_* with prior values τ*_AMPA_* = 4 msec, τ*_NMDA_* = 100 msec, τ*_GABA_* = *16* msec and τ*_KIR_* = 18 msec. Crucially each of these channels – as well as a leaky potassium current (*K_L_*) – had scale parameters that controlled their relative influence on the polarization of the postsynaptic membrane: *α*_*NA*,_ *α*_*Cl*,_ *α*_*CA/NA*,_ *α*_*KL*,_ and *α_KIR_* (Fig. 1C). These scale values were our key (postsynaptic) parameters of interest and were later tested for patient specific effects, compared to controls. Presynaptically, dynamics were coupled to the postsynaptic ensembles via a sigmoidal activation function, *H* (Fig. 1C), which mimics mean afferent firing rates, and depends on the membrane potential of the afferent cell population (V_pre_), the threshold firing potential (V_thresh_, set to − 40 mV), and the distribution of presynaptic firing rate. To capture the potential channel dysfunction in Patient 2; i.e., a presynaptic calcium mutation, we used the inverse of this afferent firing variance (denoted as ω, Fig. 1C) as a proxy. For this parameter, large values represented highly synchronized output, which we assume scales linearly with the availability of presynaptic calcium (Fig. 1C). This is consistent with calcium's role in the synchronization of release of neurotransmitter across active zones (Sharma and Vijayaraghavan, 2003).

Each (equivalent current dipole) source of electromagnetic signals comprised three distinct cell populations with canonical intrinsic (within source) connectivity (Moran et al., 2011c). These interacting neuronal populations represented pyramidal cells, inhibitory interneurons and granular-layer spiny-stellate cells (Fig. 1A). Sources received extrinsic inputs from other sources according to plausible anatomical criteria; with forward connections driving spiny stellate cells and backward connections driving pyramidal cells and inhibitory interneurons.

### Source localization

SPM's multiple sparse priors routine was used to identify the sources of activity in each patient's brain from their sensor-level data over the entire post-stimulus event time (0–350 msec). Specifically, averaged ERFs for standard and deviant trials were localized to 512 potential mesh points using a variational Bayesian approach following co-registration of fiducial points and sensor positions to a canonical template brain. We used the group inversion option in SPM to localize both sessions together, doing this for each patient individually (shown in Fig. 3). This approach has previously been applied to the MMN response (Moran et al., 2014). No prior constraints on source localization were used and the percent variance of sensor-level data was assessed to ensure a localization solution that accurately recapitulated the topography of sensor data (Fig. 3). Source reconstruction and subsequent DCM analysis (below) was performed using SPM12 (Wellcome Trust Centre for Neuroimaging, London, UK, www.fil.ion.ucl.ac.uk/spm).

### DCM

The MMN paradigm has been extensively studied in the context of DCM, and so sources and connectivity between regions have already been established (Garrido et al., 2008). The healthy controls used here were previously reported (Moran et al., 2014) to show responses in sources commensurate with Garrido et al. (2008). These included primary and secondary auditory sources as well as a source in the bilateral prefrontal cortex. Given that these sources were also activated in both patients (Fig. 3), a six-source model of the auditory evoked responses was specified, with the same prior locations in patients and controls (Fig. 4A).

Thalamic (stimulus bound) input was modeled with a Gaussian bump function that drove activity in bilateral Heschl's gyrus (HG; primary auditory cortex). The prior expectations of the parameters of this input represented a mean onset of 64 msec and duration of 16 msec; these were free parameters that account for subject specific differences in peripheral auditory processing. From HG, signals were passed via forward connections to superior temporal gyrus (STG), and from here to the inferior frontal gyrus (IFG) (Fig. 4A). Backward connections ensured recurrent extrinsic connections, with top-down inputs from IFG to STG and STG to HG. The source location priors were based on (Moran et al., 2014) as follows: left HG: x = − 42, y = − 22, z = 7; right HG: x = 46, y = − 14, z = 8; left STG: x = − 61, y = − 32, z = 8; right STG: x = 59, y = − 25, z = 8; left IFG: x = − 46, y = 20, z = 8; and right IFG: x = 46, y = 20, z = 8.

For our DCM analyses, MEG sensor data were fitted over 1–250 msec peristimulus time. For computation efficiency, DCMs were computed using each individual's sensor data projected using dimensionality reduction to the eight principal modes of a singular value decomposition of the data covariance, to identify the patterns with the greatest prior covariance given the lead field and source locations. This dimensionality reduction is standard in DCM for electrophysiology (cf (Litvak et al., 2011, Moran et al., 2014)). DCM optimizes a posterior density over free parameters (parameterized by its mean and covariance) via a standard variational Bayesian inversion procedure (Friston et al., 2007). In this application, we were particularly interested in the posterior estimates of channel-specific scale parameters and firing precision as proxy for presynaptic calcium.

In order to accommodate both standard and deviant conditions, we allowed for modulatory effects on all extrinsic connections (i.e., forward connections and backward connections). This has previously been shown to characterize the differences in source responses between standard and deviant tones (Garrido et al., 2007). These are often the parameters of interest in DCM studies, however here we were interested in the common neural physiology underlying both conditions (i.e., the weights of each channel on each source).

To identify parameter starting estimates, an initial DCM optimization was performed for 188 control recording sessions (94 control subjects — with 2 recording sessions). These posterior values were then used to compute the average conditional means across control subjects. This average was then used to initialize a second set of optimizations for the estimation of 192 subject-specific DCMs (188 sessions for control subjects, 4 sessions for channelopathy patients). This initialization ensured that the parameter estimate search began in the same part of parameter space for both patients and controls, and gave initial estimates appropriate to the MMN task.

We then examined the mean and variance of the estimated channel parameters. In particular, we focused on six parameters: AMPA receptor-mediated postsynaptic sodium connectivity (*α_NA_*), NMDA receptor-mediated postsynaptic calcium and sodium (*α_CA/NA_*), GABA_A_ receptor-mediated postsynaptic chloride (*α_Cl_*), postsynaptic potassium leak (*α_KL_*), postsynaptic inward-rectifying potassium (*α_KIR_*), and presynaptic calcium (ω). We hypothesized that Patient 1 (K^+^) would show a decrease in the mean posterior estimates of the inward-rectifying potassium channel and/or potassium leak, compared to the control cohort estimates and that Patient 2 (Ca^2 +^) would show a decrease in the mean posterior estimate of their presynaptic calcium channel.

## Results

### Mismatch negativity results

**Fig. 2:**
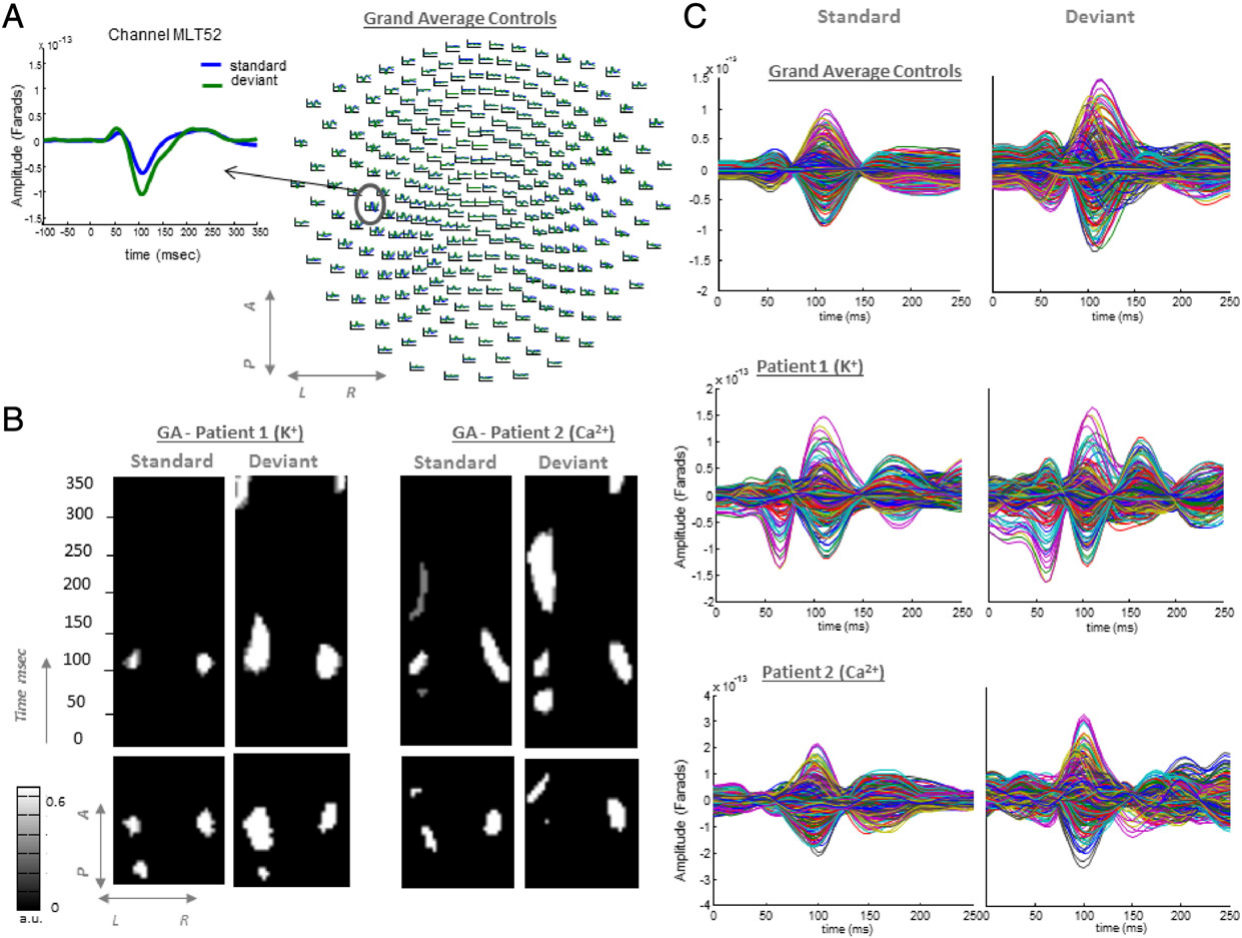
Sensor level responses to auditory tones. A. Grand averaged event related fields from the control group illustrate the mismatch negativity effect. Specifically, larger negative potentials are recorded over temporal (inset) and frontal sites for infrequent deviant tones (ERF in green) compared to responses to frequent standard tones (ERF in blue). B. Deviations from the grand-averaged control data (defined as absolute deviations > 0.1 pF) were identified for both patients. Differences (greater than 0.1 pF) are plotted from 0 to 1 in gray scale (normalized to the maximum difference, illustrated in white) for the standard and deviant tones separately, with topological effects illustrated in the bottom panel and temporal effects in the upper panels. For Patient 1, bilateral differences were observed over temporal and posterior sites around 100 msec with extended differences for deviant tones. For Patient 2 bilateral effects extended to frontal regions and were dispersed across the trial for both stimulation types. C. Butterfly plots – that show responses over all the sensors – demonstrate that controls and patients exhibit differences between standard and deviant event-related responses and were hence modeled as separate trials in the DCM, following (Moran et al., 2014). These responses were detrended using a first order discrete cosine transform.

At the MEG sensor level, we examined evoked responses to MMN standard and deviant tones to assess whether our DCM analysis was informed by an appropriate amount of experimental variance (Fig. 2). Grand-averaged data from the control cohort revealed typical differences between evoked responses to standard and deviant tones, with larger negative responses to deviant tones, maximal from 100–150 msec (Fig. 2A). Topographically, these effects were pronounced around temporal and frontal sensors bilaterally (Fig. 2A). Differences in these responses (defined as absolute deviations > 0.1 pF), between patients and controls were investigated in terms of topographic and temporal effects. These were found bilaterally at mid-temporal and posterior sensors for Patient 1 (K^+^) (Fig. 2B), with these effects centered on 100 msec and larger for deviant trials (Fig. 2B), with late effects observed in the deviant condition (Fig. 2B). Patient 2 (Ca^2 +^) exhibited similar differences focused at mid-temporal and frontal sensors, having more distributed differences temporally, with differential responses in both standard and deviant trials from 200–250 msec (Fig. 2B). In both patients and controls, differences were observed between standard and deviant tones (Fig. 2C), which were modeled as separate conditions in the DCM. In Patient 1 (K^+^) these differences were seen early (~ 50 msec), mid (~ 150 msec) and later (~ 250 msec) in the trial, while in Patient 2 (Ca^2 +^) pronounced differences were observed in mid and later evoked components (Fig. 2C).

### Source localization and source dynamics

**Fig. 3:**
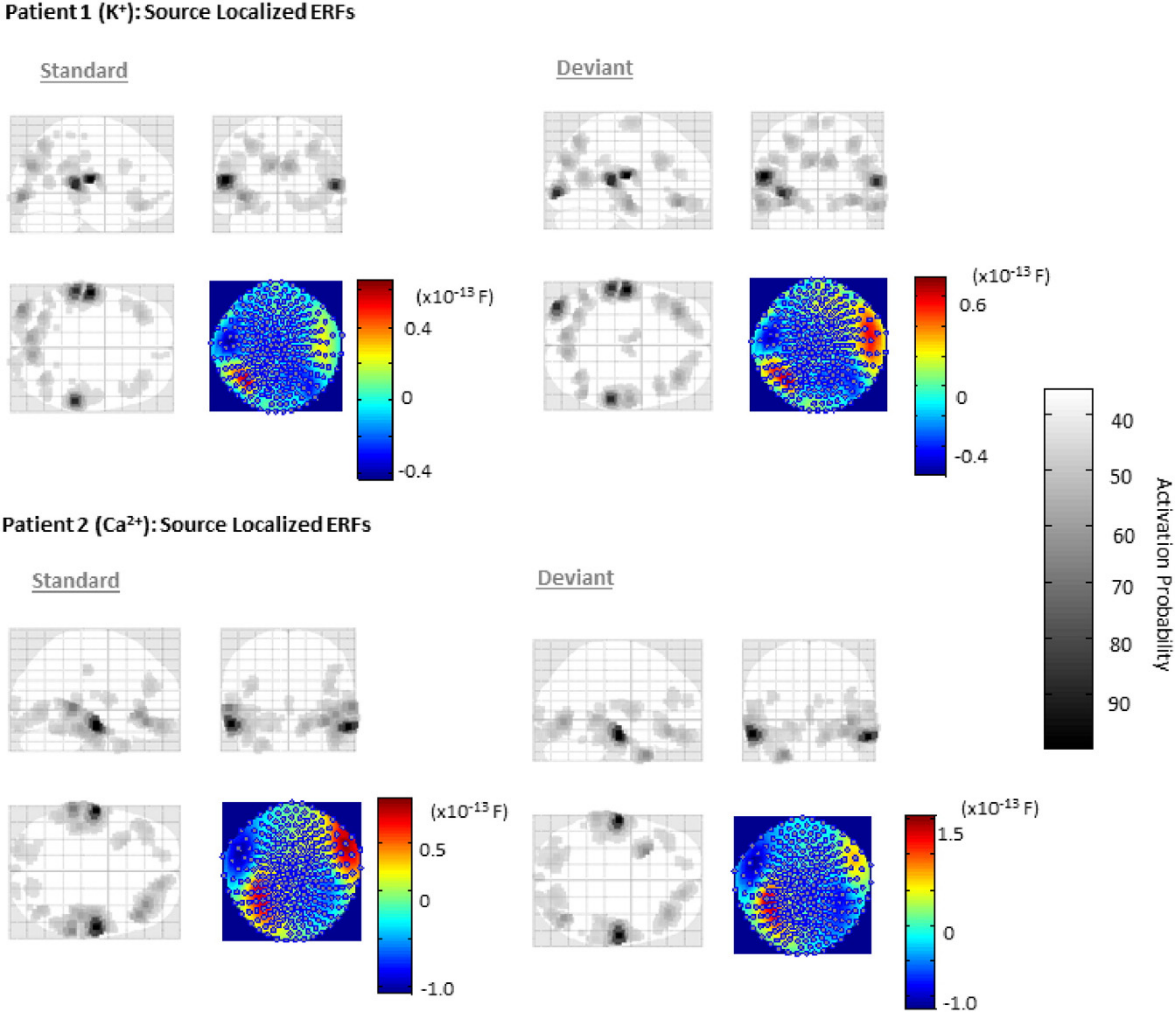
Source localized event related responses. An inverse solution constrained via a multiple sparse priors identified bilateral sources of activity in both patients at temporal and frontal sites for both trial types. Illustrated here are the MIPs (maximum intensity projection images from each subject at 100 msec). For Patient 1, 96% of the sensor data variance (jet-scale insert) was explained for both trial types using a 512-dipole mesh. For Patient 2, 95% of the sensor data was explained for both trial types using a 512-dipole mesh.

**Fig. 4:**
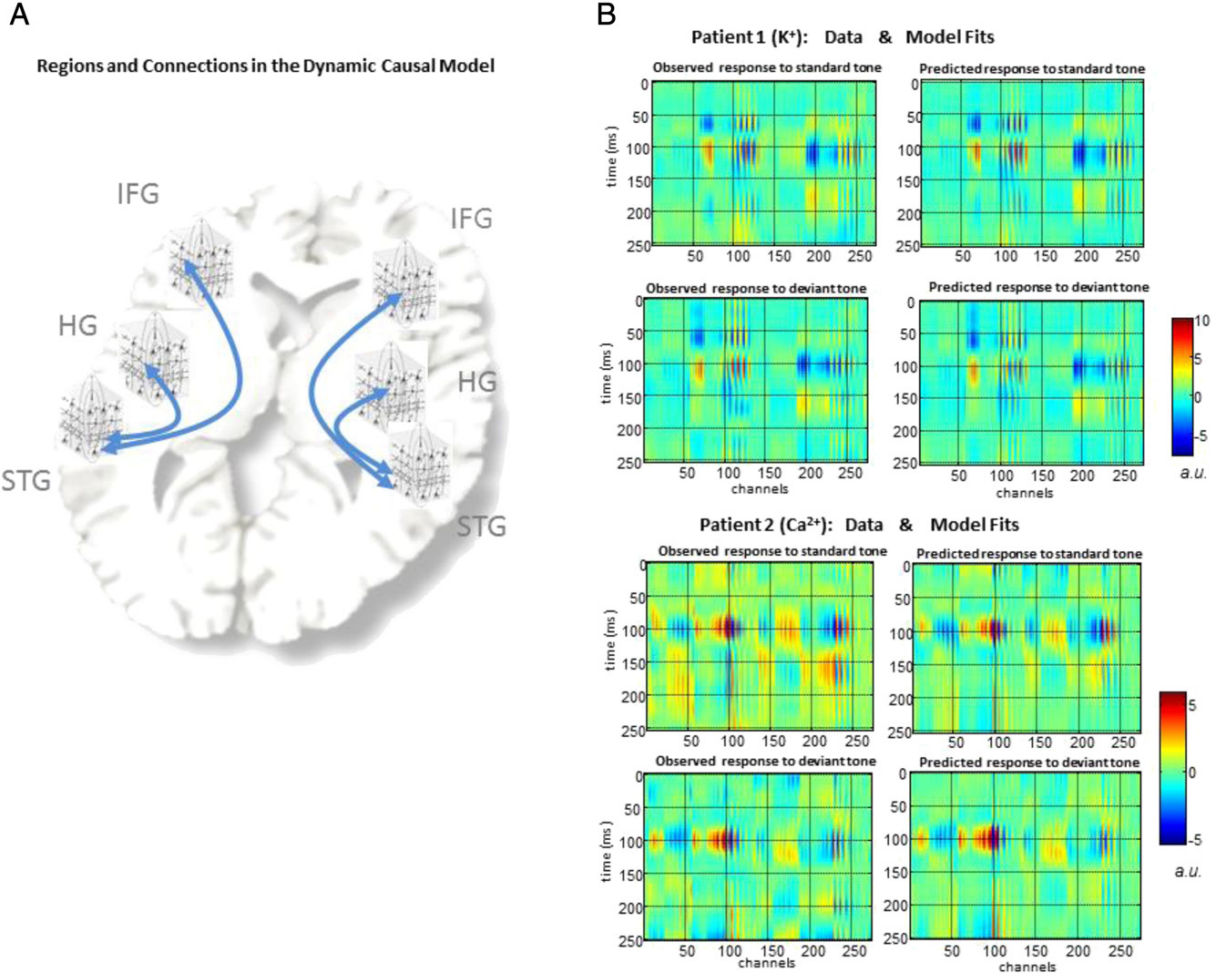
Fig. 4 optimized dynamic causal models. A. The event related fields were modeled in source space using a DCM that comprised 6 sources. These were bilateral Heschl's gyrus (HG), bilateral superior temporal gyrus (STG), and bilateral inferior frontal gyrus (IFG). Sources were connected reciprocally with forward connections from HG to STG and STG to IFG and backward connections from IFG to STG and STG to HG within-hemisphere. B. Two dimensional images representing the sensor based data. Data are adjusted using SPM's standard confounds (spm/toolbox/dcm_meeg/spm_dcm_erp_data.m), i.e., we removed the first component of a discrete cosine transform basis set, effectively mean correcting the sensor data. On the right panels are the optimized DCM-based fits. The model recapitulated the topography and temporal features in each patient's trial-specific response.

To confirm that patient sources were commensurate with neurotypical responses (Moran et al., 2014), we performed a source localization analysis on each patient and condition (i.e., standard/deviant) individually (Fig. 3). This inverse solution produces probabilistic estimates of activity on a cortical mesh. Consistent with responses in normal controls (Garrido et al., 2008), sources for both patients were obtained in bilateral temporal and inferior frontal regions. Fig. 3 presents a maximum intensity projection (MIP) image for each patient and each trial for activity at 100 msec. Here, for all 4 inverse solutions, a forward mapping to sensor space explained > 95% of variance in measured data. Given appropriate source activity, we then proceeded with a six-source DCM, comprising bilateral primary, secondary and inferior frontal regions (Fig. 4A).

From the optimized DCMs we examined the MMN response in each source (not shown). Across cell populations (i.e., excitatory spiny stellate, inhibitory interneuron, and excitatory pyramidal cells), there were no differences in the response properties to standard and oddball tones in primary auditory cortex. Consistent with previous source analyses of the MMN (Garrido et al., 2008, Moran et al., 2014), both left and right A1 showed robust responses to auditory stimuli beginning at approximately 50 msec post onset, peaking at approximately 65 msec post-onset. Within bilateral STG and IFG we found signature MMN responses (i.e., larger amplitude responses to oddball compared with standard tones) across cell population types. Bilateral STG showed robust responses to both standard and deviant tones beginning at approximately 70 msec post-onset, peaking at approximately 100 msec post-onset. Bilateral IFG followed a pattern similar to STG, though with a later onset of approximately 100 msec post-onset, peaking at approximately 150 msec post-onset. This pattern was consistent across patients and controls, suggesting that the DCMs captured the dynamic response properties of neuronal populations for patients across sources in the auditory hierarchy.

### Optimized DCMs

The goodness of our model fits were assessed in terms of percent variance explained. Specifically, we tested the variance explained of our 8-mode, reduced data space. These model fits ranged from 25.5% to 98.1% for control subjects, with an average of 83.2% of the variance explained. Patient 1 had fits of 94.9% and 94.6% of the variance explained for runs 1 and 2 respectively, while patient 2 had fits of 71.1% and 82.9% respectively. In short, the DCMs, recapitulated both healthy and diseased responses using the model described above, with an average percent variance explained amongst controls of 83.2% (SD = 0.1%) and patients of 85.8% (SD = 11.31%). In Fig. 4B we show the fits to empirical sensor-level data for the patients. These data were corrected using SPM's default detrend option, which applies a mean correction. Given the poorer fit of session 1 for patient 2 (71.1%), we elected to remove this session from further analysis. Thus, for patient 1 and controls we used between-session mean and variance estimates for further analyses, while for patient 2 we used single-session mean and variance estimates for further analyses.

DCM estimates a multivariate (Gaussian) posterior density over free parameters, from which we harvested both the posterior mean and variance of those ion channel parameters of interest — from both patients and from all control datasets. These included (log scale) parameters that controlled the relative influence of each individual ion channel on postsynaptic membrane depolarization, including an glutamatergic (AMPA)-mediated sodium channel: ***α_NA_***, a GABAergic (GABA_A_)-mediated chloride channel: ***α_Cl_***, an NMDA-mediated sodium/calcium channel: ***α_CA/NA_***, a leaky potassium channel ***α_KL_***, and an inward-rectifying potassium channel ***α_KIR_*** (Fig. 1C). We also examined the presynaptic calcium parameter, **ω**.

Using these parameters of interest, we quantified the within-subject stability of controls' parameter estimates from session 1 to session 2 using the intraclass correlation coefficient (ICC). The ICC describes how strongly two measurements in the same grouping (here, within-subject parameter estimates) resemble each other. Therefore, we directly compared session 1 and session 2 parameter estimates. To control for outliers, we measured the maximum posterior covariances between our six parameters of interest and all of the remaining parameters individually. We then removed control subjects whose DCM estimates showed high covariance between the parameter of interest and all other parameters, retaining 90% of control participants' data. Using this criterion, reliable parameter estimates from session 1 to session 2 were found for both the presynaptic calcium (r = 0.29, F = 1.8, p < 0.01) and leaky potassium (r = 0.31, F = 1.91, p < 0.01) parameters. In addition, the inward-rectifying potassium parameter estimate was found to be reliable from session 1 to 2 using a less stringent criterion (r = 0.18, F = 1.44, p = 0.05). The receptor-mediated parameters demonstrated lower between-session stability measures (AMPA-mediated sodium parameter: r = 0.05, F = 1.11, p = 0.12; GABA_A_-mediated chloride parameter: r = 0.14, F = 1.33, p = 0.12; NMDA-mediated sodium/calcium parameter: r = 0.144, F = 1.34, p = 0.11). These findings suggest moderate to high levels of consistency between sessions for our control group.

Having established the accuracy of DCM estimates, we then addressed our hypotheses from two points of view. First, we asked whether there were any significant differences between the two patients and the control group using classical inference at the between-subject level. Second, we asked the more pragmatic question of whether the parameter estimates from DCM could be used to classify the patients based upon their known pathophysiology (i.e., Patient 1: K^+^ postsynaptic inward-rectifying ion channel mutation; Patient 2: presynaptic Ca^2 +^ ion channel mutation).

### Detecting pathology

Our first analysis used the standard summary statistic approach to summarize each participant with the posterior expectations of the parameters of interest (coefficients scaling channel-specific dynamics: ***α_NA_***, ***α_Cl_***, ***α_CA/NA_***, ***α_KL_***, ***α_KIR_***, **ω**, Fig. 1C). To do this, we averaged the parameter estimates over both sessions (to remove within-subject variance) and used canonical variates analysis (CVA) to test the null hypothesis that the parameter estimates from the patients and control group were drawn from the same distribution. CVA can be thought of as a simple extension of ANOVA that accommodates multivariate response variables; here, the six parameters of interest. Our explanatory variables (or design matrix) included the patient effect relative to the controls, and an average control effect. The significance of differences between patient and control group were assessed for each patient separately using the appropriate transform of Wilks Lambda. Differences between the patient and group were tested with an *F* contrast. This analysis identified two significant canonical variates, confirming a multi-dimensional independence between the control group and patients (Patient 1: r = 0.96, p < 0.01; Patient 2: r = 0.96, p < 0.01), i.e., they were not drawn from the same distribution. As a first pass, these findings suggest that the DCM parameter estimates vary between patients and control participants, suggesting unusual signaling dynamics for our two patients.

Having determined that the six estimated parameters of interest varied between patients and controls, we then tested specificity for control data across channels using a leave-one-out cross-validation, where a control parameter was removed from the average density and compared to the average density from the remaining participants. This analysis allowed us to determine whether specific channels within our large control sample could be classified as exhibiting unusual ion channel parameter estimates. At a threshold of P > 0.90 probable difference level, this procedure yielded a true negative rate of 95.7% for rectifying potassium and 93.6% for our assay of presynaptic calcium. In addition, specificity was tested for the receptor systems of non-assayed channels, and returned a true negative rate of 89.3% for leaky potassium, 96.8% for the NMDA receptor-mediated sodium/calcium channel, 94.6% for the AMPA receptor-mediated sodium channel, and 94.6% for the GABA_A_ receptor-mediated chloride channel. Thus, this analysis suggests high specificity rates for detecting negative results in our control group using the posterior estimates from our parameters of interest.

### Detecting specific channel mutations

We then asked how sensitive the patient estimates from the parameters of interest were in detecting aberrant dynamics. Specifically we computed the average control probability density and compared this to the conditional density from each patient separately. We used the non-overlapping probability density to quantify the difference in parameter estimates. Here, we successfully identified a mutated potassium channel – the leak potassium channel (***α_KL_***) – as abnormal in Patient 1 with a sensitivity of 100%. In addition to this channel, the test also showed abnormality in the AMPA receptor-mediated sodium channel (***α_NA_***) and the presynaptic calcium channel (**ω**). No other channels showed sensitivity differences for Patient 1. For Patient 2, this same analysis did not identify abnormal parameter estimates in the mutated presynaptic calcium channel, however the GABA_A_ receptor-mediated channel parameter estimates (***α_Cl_***) were identified as abnormal. No other channels showed sensitivity differences for Patient 2. These findings suggest that the DCM parameter estimates alone point to abnormal ion channel signaling dynamics in both patients. Though there is a stronger case for identification of the pathological channel in Patient 1 than Patient 2, Patient 2 exhibited enhanced hyperpolarizing currents, consistent with reduced synaptic transmission characteristic of this mutation's physiological effect (Pietrobon, 2005). Importantly, Patient 1 had a mutation in their postsynaptic channel, and these dynamics are modeled to a much greater extent than presynaptic dynamics (Fig. 1C). For our analysis, we used a measure of afferent firing variance as a proxy for presynaptic calcium. However, we did not add a specific ion channel denoting presynaptic calcium to our model. This might account for the lack of identification of presynaptic calcium signaling difficulties for Patient 2, though the test did identify differences in at least one ion channel.

### Diagnostic implementation

**Fig. 5:**
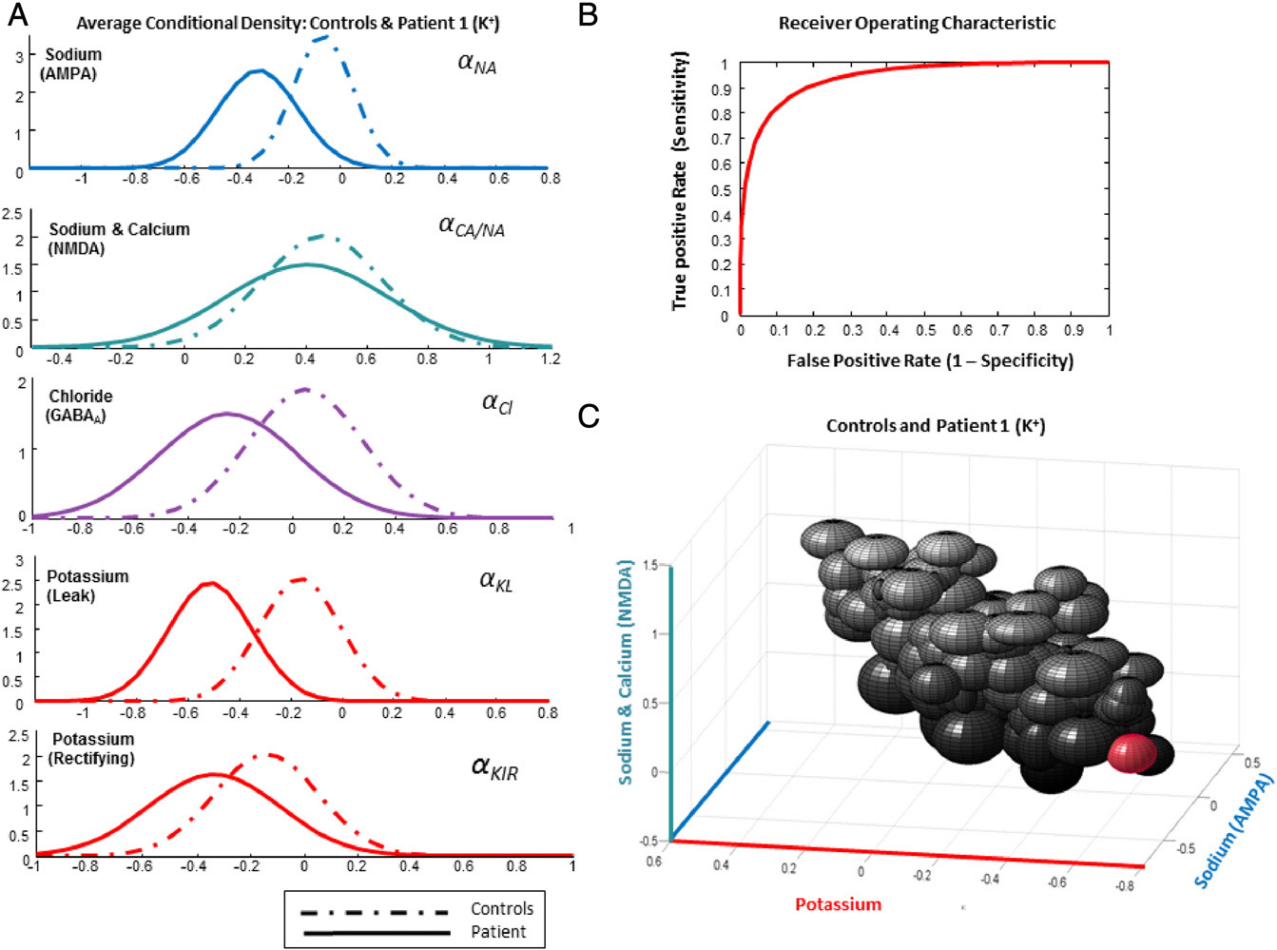
Patient 1 (K^+^) vs. Controls, DCM conditional parameter estimates. A. DCM channel estimates: illustrated are the controls' average Gaussian probability density function (PDF) — computed using the overall subject mean and between-subject variability as the mean and variance of the control group respectively. Individual channel estimates are plotted against the average estimate of Patient 1, where the patient PDF used the average within-subject variability from the controls as a measure of patient variance. B. Receiver operating characteristic demonstrating the sensitivity and specificity of the test on the potassium leak parameter. By altering the decision threshold from the midpoint of the population means (from one-tenth to twice this value), the area under the curve (AUC) was found to be 0.94. C. Ellipsoids (Bayesian confidence regions) drawn to represent three posterior channel weights (AMPA mediated sodium, NMDA mediated sodium/calcium and leak potassium) from each DCM with Patient 1 (K +) in red. Consistent with the average probability statistic in A, the patient is an outlier on the negative side of the potassium channel axis (red).

**Fig. 6:**
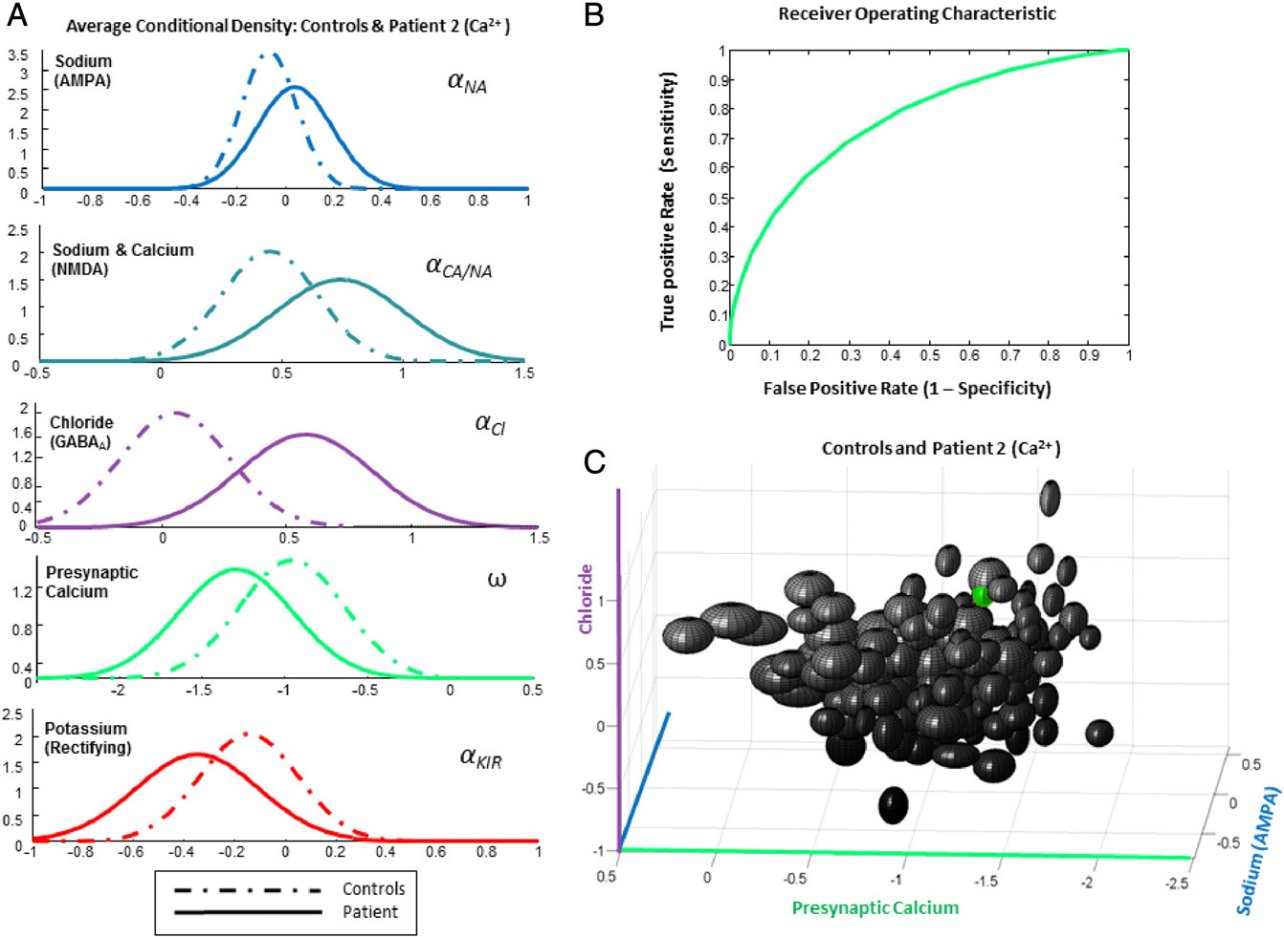
Patient 2 (Ca^2 +^) vs. Controls, DCM conditional parameter estimates. A. As in Fig. 5, we compared the average probability density across channel parameters from the control cohort (dashed lines) to the second calcium channelopathy patient with a presynaptic calcium mutation (full lines). B. Receiver operating characteristic demonstrating the sensitivity and specificity of the test on the presynaptic calcium channel parameter with an AUC = 0.76. C. Ellipsoids (Bayesian confidence intervals) drawn to represent three posterior channel weights (AMPA-mediated sodium, GABA-mediated chloride and presynaptic calcium) from each DCM with Patient 2 (Ca^2 +^) identified in green. This patient was classified as abnormal along the chloride channel dimension.

As a further analysis strategy, we addressed the utility of the parameter estimates in classifying patients. Although we only had one patient diagnosed with mutations in each ion channel, it was possible to construct receiver–operator curves characterizing the sensitivity and specificity of classification. For this we constructed a control average Gaussian probability density function using the overall subject mean and between-subject variability as the mean and variance of the control group respectively. For the patients we used the average within-subject variability from the controls (i.e., variance between the two sessions) as a measure of patient variance, along with the estimated patient mean. These are illustrated for controls versus Patient 1 in Fig. 5A and for controls versus Patient 2 in Fig. 6A. We then computed a receiver operating characteristic (ROC) for the postsynaptic potassium leak parameter, ***α_KL_***, as well as for the presynaptic calcium parameter, **ω**. In other words, we examined sensitivity and specificity for mutated parameter estimates using a decision threshold from the midpoint of controls versus patients and scaled the decision threshold from one tenth to twice this value (Johnson, 2004). The resulting ROC curves were summarized with their area under the curve (AUC). A value of 1 means perfect sensitivity for all levels of specificity, while a value of a half represents a poor test where sensitivity is equal to specificity. The ability of each mutated ion channel parameter to discriminate between patients and controls was assessed separately. For Patient 1 compared to controls, the leaky potassium parameter ***α_KL_***, produced an AUC of 0.94 (Fig. 5B) and for Patient 2 compared to controls, the presynaptic calcium parameter estimate **ω** produced an AUC = 0.76 (Fig. 6B). Clearly, these analyses do not represent a true cross-validation (because we only had one patient for each patient group); however they represent a quantitative assessment of the classification performance that could, in principle, be obtained given the within and between-subject variances in parameter estimates.

In order to illustrate the selective nature of these parameter differences, we plotted the Bayesian confidence interval (posterior mean plus and minus one standard deviation) as an ellipsoid for three ion channel parameters for every control session set and for each patient (Figs. 5C, 6C). As shown in Figs. 5C and 6C, the patients can be seen as outliers along the potassium dimension for Patient 1 (***α_KL_***) (red ellipsoid, Fig. 5C) and along the chloride/presynaptic calcium dimensions for Patient 2 (***α_Cl_*/ω**) (green ellipsoid, Fig. 6C). The ellipsoid plot for Patient 1 clearly demonstrates that their leaky potassium parameter estimate represents an outlier from controls. The ellipsoid plot of Patient 2 also demonstrates that their GABA_A_-mediated chloride parameter estimate is an outlier, while their presynaptic calcium parameter estimate lies in the negative quartile.

## Discussion

These results demonstrate that biophysical models of synaptic channels can be estimated using non-invasive magnetoencephalographic responses to simple auditory stimuli. Moreover these DCM results highlight that the model of synaptic activity has validity in relation to underlying neurobiological processes, with abnormal posterior ranges identified for ion channels concordant with genetic mutations in two patients with distinct channelopathies.

At present, the diagnosis, prognosis and treatment of ion channelopathies is impeded by the phenotypic heterogeneity of disease manifestation, the necessity of a high degree of clinical suspicion, and significant time delays associated with confirming test results (Cannon, 2006, Hanna, 2006, Kullmann, 2010). In addition, mounting evidence points to the likelihood that polygenic channel dysfunction underlies disorders considered until now idiopathic (Cannon, 2006, Hanna, 2006). Finally, autoimmune channelopathies, a special case of acquired channelopathies, can present with diverse clinical symptoms, including encephalitis accompanied by psychiatric symptoms (Gable et al., 2009), sleep disturbances (Tsutsui et al., 2012), and even memory impairments (Shimazaki et al., 2007). These diverse clinical presentations render it desirable for clinicians to have an inexpensive tool — offering a first-pass means of narrowing the range of diagnoses or directing genetic sequencing efforts. The developments presented here offer promise for such an assay, given that mismatch auditory responses are easily observed in EEG data, collected routinely in clinical settings. Importantly, DCMs with similar ion channel parameterization and regional distribution have previously been applied and tested using EEG-generated auditory evoked responses (Boly et al., 2011). Such an in vivo strategy would also aid in clinical diagnosis by providing a functional assay which incorporates putative compensatory and environmental interactions with the underlying channel dysfunction.

DCM is a modeling framework that provides a mechanistic estimation of how measured signals (here, cortical electrophysiological) are generated. To date, clinical assessments of channelopathies in vivo have focused on peripheral electrophysiological abnormalities measured using electromyography and nerve conduction analysis (Tomlinson et al., 2009). Motivated by epilepsy coincidence, the EEG has also been characterized as abnormal in patients similar to our Patient 2, with mutations in CACNA1A gene, where mixed patterns of slowing and spike and wave activity have been observed in scalp potentials (Chan et al., 2008). Our approach differs from these analyses by eschewing examination of morphological characteristics of patient data directly. Indeed, our findings demonstrate that sensor and source-level evoked responses show a characteristic MMN response to standard and deviant tones in patients as well as controls. However DCM models the waveform in its entirety, and does not rest on topological or trial differences. Rather a full generative model – designed to capture contributions from different ion channels with distinct time constants and voltage or neurotransmitter gating — is applied to explain multichannel responses over all peristimulus time.

The models we apply have been motivated by in vitro characterizations of neuronal channel dynamics. As proof-of-principle that DCM can identify abnormal dynamics with some channel specificity, we have applied an augmented neural mass model to patients with known ion channel signaling dysfunction. Previous in vitro characterizations of cells with mutated channels have demonstrated a net loss of function associated with presynaptic calcium and potassium channel gene mutations, leading to impaired high-frequency spiking (Plomp et al., 2009) and impaired repolarization respectively (Jongsma and Wilders, 2001). Importantly, given just ERFs collected from non-invasive electrophysiological recordings our models identify probabilistic abnormalities in estimated parameters encoding these types of cellular mechanisms. While demonstrating exact specificity of the DCM channel estimates will require larger samples, our results demonstrate that different synaptic parameters are associated with distinct mutations in a direction commensurate with physiological effects measured in vitro. Interestingly in Patient 1, our model identified abnormality in the leak potassium (*KL*) rather than the rectifying potassium channel (*KIR*). However, functionally both channels operate with the same basic I–V characteristic and with identical reversal potentials but with *KIR* exhibiting higher conductance than *KL* at highly hyperpolarized states (Shen et al., 2007). Our choice of dynamic parameterization — which gated this *KIR* channel to operate only at hyperpolarized states (Fig. 1C), may account for this apparent discrepancy since our model weighted contributions from only leak potassium at depolarization above − 75 mV. Put another way, it is likely that both channels have highly similar dynamic characteristics in the voltage range excited by our task, and perhaps cannot be dissociated by the DCM (Shen et al., 2007).

While our model of rectifying potassium may be slightly over-constrained, our model's parameterization of presynaptic calcium is most likely under-constrained. This is reflected in its poor performance in terms of diagnostic sensitivity and specificity. Previous work has shown that this parameter can produce profound effects on the characteristics of an evoked response (Moran et al., 2011a) and is also likely to reflect activity of other ‘gain control’ mechanisms like classical neuromodulatory effects at cholinergic receptors (Moran et al., 2013b). We used the afferent firing variance in our DCM model as a proxy for a measure of presynaptic calcium, and we assumed that highly synchronized values presynaptically would scale linearly with the amount of presynaptic calcium. However, our results indicate that this measure did not show sensitivity in Patient 2. Thus, our model would benefit from more constraints presynaptically, in order to accurately reflect specific ion channel signaling dynamics of the presynaptic membrane. However the physiological loss-of-function seen in genetic CACNA1A knock-out animals is commensurate with the postsynaptic channel effect we observed in this patient (Pietrobon, 2005).

In terms of a clinically pragmatic diagnostic test, we tested two 6-minute sessions from each patient. This was important given that one session in Patient 2 produced poor model fits (~ 70% variance explained). However, considering an either/or approach, at least one session was optimized to levels consistent with those of control DCMs, suggesting there was no bias in model fits due to ion channel signaling differences between groups. Overall, these findings indicate DCM offers an effective tool for determining specific postsynaptic ion channel signaling deficits in vivo, and that more constraints on presynaptic ion channel signaling could lead to a similarly precise measurement. It is important to note that we have modeled pathological ion channel signaling using patients with known genetic defects in specified ion channels. It remains to be seen whether our assay could be applied to patients with potentially unknown ion channel signaling pathology and whether the type of DCMs presented here could be used to identify the underlying abnormalities. For example, heterogeneous channel dysfunction is hypothesized to underlie a range of psychiatric and neurological disorders as diverse as schizophrenia (Jardri and Deneve, 2013) and migraine (Chan et al., 2008). Recent research has applied DCM to measure the role of systemic excitatory and inhibitory intrinsic connectivity in seizures related to NMDA-receptor encephalitis (Cooray et al., 2015). In this autoimmune disorder, symptoms of psychosis often precede seizure activity. However, diagnosis via EEG using traditional metrics does not provide the specificity of a DCM-type investigation. It is conceivable that the type of analysis we demonstrate here could be applied in an ambulatory setting, to test for a pathological antibody cause. In this vein, research using DCM for electrophysiology has begun to explore the role of NMDA and cholinergic channel dysfunction in the development of schizophrenia using a ketamine model in both animal (Moran et al., 2015) and human (Moran et al., 2015, Schmidt et al., 2012, Schmidt et al., 2013) studies. These studies converge on altered plasticity mechanisms related to NMDA receptor activity, and further suggest an aberrancy (hyperexcitability) of fast synaptic responses that may impede learning. Together these studies demonstrate two advantages of DCM. First, they hold promise for uncovering unknown channel pathology in complex diseases. Second, they provide a platform where animal studies can be complemented with human investigations using identical analysis and parametric frameworks. Given the high sensitivity and specificity found here, we are optimistic that the models of synaptic function can be combined with more developed model comparisons to quantify subtle differences in the synaptic effectors of a particular clinical patient presentation. For example, we envision that the parameter setting identified in our control population could serve as a new set of prior parameters for DCM and that single or multiple parameter differences from normal could be tested using Bayesian model comparison and reduced model space.

We have illustrated how a biophysically informed analysis like DCM allows for the characterization of recorded signals, in terms of clinically meaningful model parameters, with representations of specific ion channels that mediate neuronal dynamics (Moran et al., 2011c). While we know that ‘ground truth’ in EEG and MEG are those data recorded directly from electrodes and sensors, a neural mass model analysis like DCM allows a richer characterization of the recorded signal, with representations of specific ion species and the channels by which they subserve neural communication (Moran et al., 2011b). Interrogating data with highly constrained models thus allows an efficient estimation of key model parameters that are potentially very useful in a clinical setting — i.e., parameters that can be linked mechanistically to known pathophysiological mechanisms. In conclusion, the refinement of model parameters of ion channel dynamics in DCM for electrophysiology could ultimately lead to the use of EEG or MEG measurements as a novel adjunct in the treatment of patients with suspected ion channel dysfunction.

## References

[bb0005] Boly M., Garrido M.I., Gosseries O., Bruno M.A., Boveroux P., Schnakers C., Massimini M., Litvak V., Laureys S., Friston K. (2011). Preserved feedforward but impaired top-down processes in the vegetative state. Science.

[bb0010] Boly M., Moran R., Murphy M., Boveroux P., Bruno M.A., Noirhomme Q., Ledoux D., Bonhomme V., Brichant J.F., Tononi G. (2012). Connectivity changes underlying spectral EEG changes during propofol-induced loss of consciousness. J. Neurosci..

[bb0015] Cannon S.C. (2006). Pathomechanisms in channelopathies of skeletal muscle and brain. Annu. Rev. Neurosci..

[bb0020] Catterall W.A., Dib-Hajj S., Meisler M.H., Pietrobon D. (2008). Inherited neuronal ion channelopathies: new windows on complex neurological diseases. J. Neurosci..

[bb0025] Chan Y.-C., Burgunder J.-M., Wilder-Smith E., Chew S.-E., Lam-Mok-Sing K.M., Sharma V., Ong B.K. (2008). Electroencephalographic changes and seizures in familial hemiplegic migraine patients with the *CACNA1A* gene S218L mutation. J. Clin. Neurosci..

[bb0030] Cooray G.K., Sengupta B., Douglas P., Englund M., Wickstrom R., Friston K. (2015). Characterising seizures in anti-NMDA-receptor encephalitis with dynamic causal modelling. NeuroImage.

[bb0035] Dager S.R., Oskin N., Richards T.L., Posse S. (2008). Research applications of magnetic resonance spectroscopy (MRS) to investigate psychiatric disorders. Top. Magn. Reson. Imaging.

[bb0040] David O., Kilner J.M., Friston K.J. (2006). Mechanisms of evoked and induced responses in MEG/EEG. NeuroImage.

[bb0045] Friston K., Mattout J., Trujillo-Barreto N., Ashburner J., Penny W. (2007). Variational free energy and the Laplace approximation. NeuroImage.

[bb0050] Friston K.J., Harrison L., Penny W. (2003). Dynamic causal modelling. NeuroImage.

[bb0055] Gable M., Gavali S., Radner A., Tilley D., Lee B., Dyner L., Collins A., Dengel A., Dalmau J., Glaser C. (2009). Anti-NMDA receptor encephalitis: report of ten cases and comparison with viral encephalitis. Eur. J. Clin. Microbiol. Infect. Dis..

[bb0060] Garrido M.I., Friston K.J., Kiebel S.J., Stephan K.E., Baldeweg T., Kilner J.M. (2008). The functional anatomy of the MMN: a DCM study of the roving paradigm. NeuroImage.

[bb0065] Garrido M.I., Kilner J.M., Kiebel S.J., Friston K.J. (2007). Evoked brain responses are generated by feedback loops. Proc. Natl. Acad. Sci. U. S. A..

[bb0070] Hanna M.G. (2006). Genetic neurological channelopathies. Nat. Clin. Pract. Neurol..

[bb0075] Helbig I., Scheffer I.E., Mulley J.C., Berkovic S.F. (2008). Navigating the channels and beyond: unravelling the genetics of the epilepsies. Lancet Neurol..

[bb0080] Jardri R., Deneve S. (2013). Circular inferences in schizophrenia. Brain.

[bb0085] Johnson N.P. (2004). Advantages to transforming the receiver operating characteristic (ROC) curve into likelihood ratio co‐ordinates. Stat. Med..

[bb0090] Jongsma H.J., Wilders R. (2001). Channelopathies: Kir2. 1 mutations jeopardize many cell functions. Curr. Biol..

[bb0095] Klassen T., Davis C., Goldman A., Burgess D., Chen T., Wheeler D., McPherson J., Bourquin T., Lewis L., Villasana D. (2011). Exome sequencing of ion channel genes reveals complex profiles confounding personal risk assessment in epilepsy. Cell.

[bb0100] Kullmann D.M. (2010). Neurological channelopathies. Annu. Rev. Neurosci..

[bb0105] Lee C.-M., Farde L. (2006). Using positron emission tomography to facilitate CNS drug development. Trends Pharmacol. Sci..

[bb0110] Litvak V., Mattout J., Kiebel S., Phillips C., Henson R., Kilner J., Barnes G., Oostenveld R., Daunizeau J., Flandin G., Penny W., Friston K. (2011). EEG and MEG data analysis in SPM8. Comput. Intell. Neurosci..

[bb0115] Marreiros A.C., Kiebel S.J., Daunizeau J., Harrison L.M., Friston K.J. (2009). Population dynamics under the Laplace assumption. NeuroImage.

[bb0120] Moran R., Pinotsis D.A., Friston K. (2013). Neural masses and fields in dynamic causal modeling. Front. Comput. Neurosci..

[bb0125] Moran R.J., Campo P., Symmonds M., Stephan K.E., Dolan R.J., Friston K.J. (2013). Free energy, precision and learning: the role of cholinergic neuromodulation. J. Neurosci..

[bb0130] Moran R.J., Jones M.W., Blockeel A.J., Adams R.A., Stephan K.E., Friston K.J. (2015). Losing control under ketamine: suppressed cortico-hippocampal drive following acute ketamine in rats. Neuropsychopharmacology.

[bb0135] Moran R.J., Stephan K.E., Dolan R.J., Friston K.J. (2011). Consistent spectral predictors for dynamic causal models of steady-state responses. NeuroImage.

[bb0140] Moran R.J., Symmonds M., Dolan R.J., Friston K.J. (2014). The brain ages optimally to model its environment: evidence from sensory learning over the adult lifespan. PLoS Comput. Biol..

[bb0145] Moran R.J., Symmonds M., Stephan K.E., Friston K.J., Dolan R.J. (2011). An in vivo assay of synaptic function mediating human cognition. Curr. Biol..

[bb0150] Moran R.J., Symmonds M., Stephan K.E., Friston K.J., Dolan R.J. (2011). An in vivo assay of synaptic function mediating human cognition. Curr. Biol..

[bb0155] Morris C., Lecar H. (1981). Voltage oscillations in the barnacle giant muscle fiber. Biophys. J..

[bb0160] Nisenbaum E.S., Wilson C.J. (1995). Potassium currents responsible for inward and outward rectification in rat neostriatal spiny projection neurons. J. Neurosci..

[bb0165] Pietrobon D. (2005). Function and dysfunction of synaptic calcium channels: insights from mouse models. Curr. Opin. Neurobiol..

[bb0170] Plomp J.J., van den Maagdenberg A.M., Kaja S. (2009). The ataxic Cacna1a-mutant mouse rolling Nagoya: an overview of neuromorphological and electrophysiological findings. Cerebellum.

[bb0175] Rubenstein J., Merzenich M. (2003). Model of autism: increased ratio of excitation/inhibition in key neural systems. Genes Brain Behav..

[bb0180] Schmidt A., Bachmann R., Kometer M., Csomor P.A., Stephan K.E., Seifritz E., Vollenweider F.X. (2012). Mismatch negativity encoding of prediction errors predicts S-ketamine-induced cognitive impairments. Neuropsychopharmacology.

[bb0185] Schmidt A., Diaconescu A.O., Kometer M., Friston K.J., Stephan K.E., Vollenweider F.X. (2013). Modeling ketamine effects on synaptic plasticity during the mismatch negativity. Cereb. Cortex.

[bb0190] Sharma G., Vijayaraghavan S. (2003). Modulation of presynaptic store calcium induces release of glutamate and postsynaptic firing. Neuron.

[bb0195] Shen W., Tian X., Day M., Ulrich S., Tkatch T., Nathanson N.M., Surmeier D.J. (2007). Cholinergic modulation of Kir2 channels selectively elevates dendritic excitability in striatopallidal neurons. Nat. Neurosci..

[bb0200] Shimazaki H., Ando Y., Nakano I., Dalmau J. (2007). Reversible limbic encephalitis with antibodies against the membranes of neurones of the hippocampus. J. Neurol. Neurosurg. Psychiatry.

[bb0205] Singh R., Scheffer I.E., Crossland K., Berkovic S.F. (1999). Generalized epilepsy with febrile seizures plus: a common childhood-onset genetic epilepsy syndrome. Ann. Neurol..

[bb0210] Tomlinson S.E., Hanna M.G., Kullmann D.M., Veronica Tan S., Burke D. (2009). Clinical neurophysiology of the episodic ataxias: Insights into ion channel dysfunction in vivo. Clin. Neurophysiol..

[bb0215] Tsutsui K., Kanbayashi T., Tanaka K., Boku S., Ito W., Tokunaga J., Mori A., Hishikawa Y., Shimizu T., Nishino S. (2012). Anti-NMDA-receptor antibody detected in encephalitis, schizophrenia, and narcolepsy with psychotic features. BMC Psychiatry.

[bb0220] Tye K.M., Deisseroth K. (2012). Optogenetic investigation of neural circuits underlying brain disease in animal models. Nat. Rev. Neurosci..

[bb0225] Yizhar O., Fenno L.E., Prigge M., Schneider F., Davidson T.J., O'Shea D.J., Sohal V.S., Goshen I., Finkelstein J., Paz J.T., Stehfest K., Fudim R., Ramakrishnan C., Huguenard J., Hegemann P., Deisseroth K. (2011). Neocortical excitation/inhibition balance in information processing and social dysfunction. Nature.

[bb0230] Yogeeswari P., Ragavendran J.V., Thirumurugan R., Saxena A., Sriram D. (2004). Ion channels as important targets for antiepileptic drug design. Curr. Drug Targets.

